# On the Connexion Between Morbid Physical and Religious Phenomena

**Published:** 1855-07-01

**Authors:** J. F. Denham


### ON THE CONNEXION BETWEEN MORBID PHYSICAL AND RELIGIOUS PHENOMENA.

No. 3 of a Series.

BY THE REV. J. F. DENHAM, M.A., F.R.S., &C.

Before tracing this connexion further by the aid of mere observation, it may be desirable to ascertain whether any sanction for our views of it can be derived from the Scriptures. It is proposed, then, to consider in this paper what the Scriptures teach respecting the body, or "the flesh," as they frequently term it, its physical qualities, and the consequences, of various kinds, produced on the mind, soul, or spirit, by its union with these intellectual and emotional principles of our nature.

The Scriptures begin by acquainting us with an immense deterioration inflicted upon the body and external circumstances of the first parents of the human race, and entailed upon all their posterity, and from which the most important practical consequences are both deducible by our reason, and are also fully recognised in other parts of the sacred volume. In the second chapter of the book of Genesis, it is stated that God formed man out of the dust of the ground in the *country* of Eden, and by breathing into his nostrils the breath of *lives,* made man a living soul; and afterwards took him and put him into the *garden* of Eden to dress it and to keep it; and that out of the ground of that garden grew every tree that is pleasant to the sight and good for food, and the tree of lives also in the midst of the garden, and the tree of the knowledge of good and evil:—that out of the man in this perfect physical state of his nature, and placed amid such favourable external circumstances, woman was formed:—that they were both naked and not ashamed:—and in the first chapter, which is a previous summary of the narrative, that God looked upon all things that he had made, the human male and female in his own image and after his own likeness included, and blessed them; and that everything seemed in the view of his infinite perceptions to be "very good," proper, and happy. In the third chapter, both the physical constitution and external condition, as well as the moral state and enjoyment, of the human species are represented as undergoing a great and adverse change. For, in consequence of their transgression, they have now become conscious of the shame of nakedness, of guilt, and fear—enmity between them and the serpent is instituted, the woman's sorrow and her conception are greatly multiplied, her will and wish are subjected to those of her husband, the ground is cursed, with at least an exuberance of troublesome vegetation, man is doomed to eat his bread in sorrow from it, in the sweat of his brow, all the days of his life, till by a chronic dissolution ("dying thou shalt die," margin) he should return to his original dust. He is driven out of the garden "to till the ground" of the country of Eden, out of which he had been taken, debarred access to the tree of life by cherubim and a flaming sword; and the very species of his future food was altered from "every herb bearing seed, which is upon the face of all the earth, and every tree in the which is the fruit of a tree yielding seed,"* to "the herb of the field."† Now it is impossible not to conclude, according to all our present observation and experience respecting the effects of physical causes upon the mental and moral constitution of man, but that these great changes in the case of our first parents, and in regard of all those physical causes which chiefly affect our nature, must have produced the most extensive alterations on their mind and moral dispositions; and these changes and their effects being transmitted to all their descendants, fully prepare us for the subsequent records, and for the existing phenomena of the perturbed state of the mental and moral nature of man. It does not appear that any change was inflicted *directly* on either the intellect or the moral affections of human nature, but these remaining in their original state, we see sufficient in the indirect effects produced upon them by means of the alteration in man's physical state and circumstances, to account not only for the moral but even mental disturbances which we perpetually experience and observe even under the most favourable physical circumstances, and for those still greater disturbances in proportion as those physical circumstances become by any means whatever still further removed from their normal condition. The well known power of such circumstances to pervert the mind and dispositions of mankind is thus afterwards described by Moses in regard of the effects of famine. "Thou shalt eat the fruit of thine own body, the flesh of thy sons and of thy daughters, which the Lord thy God hath given thee, in the siege, and in the straitness, wherewith thine enemies shall distress thee: so that the man that is tender among you, and very delicate, his eye shall be *evil* toward his brother, and toward the wife of his bosom, and toward the remnant of his children which he shall leave: so that he will not give to any of them of the flesh of his children whom he shall eat: because he hath nothing left him in the siege, and in the straitness, wherewith thine

* Gen. i. 29. † Chap. iii. 18.

enemies shall distress thee in all thy gates. The tender and delicate woman among you, which would not adventure to set the sole of her foot upon the ground for delicateness and tenderness, her eye shall be *evil* toward the husband of her bosom, and toward her son, and toward her daughter, and toward her young one (see the margin) that cometh out from between her feet, and toward her children which she shall bear: for she shall eat them for want of all things secretly in the siege and straitness wherewith thine enemy shall distress thee in thy gates."* This passage affords a specimen of the solution furnished by the Scriptures themselves of the mental and moral alienation naturally resulting from the pressure of physical circumstances. Before adducing other and more comprehensive specimens of it, it may be remarked that the Hebrew writers and their Greek translators used the words denoting, or relating to, the body, mind, soul, and spirit of man, so promiscuously, and even interchangeably, as, in the opinion of a learned and pious prelate of our church, to render it "doubtful whether they had any word ever standing for a purely immaterial principle in man," and that by their referring intellectual perceptions to the heart לב Cor, טחות præcordia, כבד jecur, כליות renes, מעים viscera, *καρδία, θυμὸς, νοῦς, φρὴν σπλάγχνα,* they at least intimated the close community between what we now call the material and immaterial parts of our nature,† and with perfect consistency, therefore, represent the sympathy between the body, soul, and spirit to be most intense and pervasive.‡ We pass over a multitude of incidental references to that sympathy in the Old Testament. In the apocryphal books we find the following allusions to it: "For the thoughts of mortal men are miserable (margin, fearful). "For the corruptible body presseth down the soul, and the earthly tabernacle weigheth down the mind that museth upon many things."§ "Great travail is created for every man, and a heavy yoke is upon *the sons of Adam*, from the day they go out of their mother's womb, till the day that they return to the mother of all things. Their imagination of things to come, and the day of death, their thoughts, and fear of heart; from him that sitteth on a throne of glory, unto him that is humbled in dust and ashes; from him that weareth purple and a crown unto him that is clothed with a linen frock. Wrath and envy, trouble and unquietness, fear of death, and anger, and strife; and in the time of rest upon his bed do change his knowledge. A little or nothing is his rest, and afterward he is in his sleep, as in a day of keeping watch, troubled in the vision of his heart, as if he was escaped out of a battle. When all is safe he awaketh, and marvelleth that the fear was nothing. Such things happen unto all flesh, and is *sevenfold more upon sinners*."|| In St. John's gospel the distinction is made, "born not of blood, nor of the will of the flesh, but of God."¶ "That which is flesh is flesh, and that which is spirit is spirit."** "Ye must be born again," and our Lord tells us that even the righteous shall become "children of God by being the children of the resurrection."†† He makes the following extensive admission in excuse for his disciples when, in the garden of Gethsemane, "he found them sleeping for sorrow, for their eyes were heavy," "the spirit indeed is willing, but the flesh is weak."‡‡

We now proceed to the examination of St. Paul's ideas and instructions respecting "the flesh and spirit," and the consequences of their union, as these are developed in his epistles, and by taking his writings in their probable chronological order. In his epistle to the Galatians, "flesh and blood" is the

* Deut. xxviii. 53—58; comp. 2 Kings vi. 28, &c.

† Bp. Law, "Theory of Religion," London, 1820, 423, 424.

‡ See Jer. iv. 19; Is. xv. 5; xvi. 11; xxi. 3, &c.

§ Wisdom of Sol. ix. 14, 15. || Ecclus. xl. 1—8.

¶ Chap. i. 13. ** Chap. iii. 6. †† Luke xx. 36.

‡‡ Matt. xxvi. 41.

collective expression for the origin of everything opposed to the better dictates of our nature. He "confers not with it," declares that "the flesh lusteth against the spirit, and the spirit against the flesh, that they are contrary the one to the other, so that we cannot fully do the things that we would;" and among the works of the flesh not only includes the more obvious sensualities, but even "idolatry, witchcraft, (or perhaps spiritual sorcery, under the term poisonings), hatred, variance, emulations, wrath, strife, seditions, heresies, envyings;" he asserts that "they that are Christ's have crucified the flesh with the passions thereof," that "he that soweth to the flesh shall of the flesh reap corruption, but he that soweth to the spirit shall of the spirit reap life everlasting." In his first epistle to the Corinthians, "the wise man after the flesh" rejects the gospel; carnal is opposed to spiritual, and carnal means "walking as men:" it is "by the destruction of the flesh that the spirit is saved in the day of the Lord Jesus." He himself "keeps under his body, and brings it into subjection, lest by any means when he had preached to others, he himself should be a cast-away." In his discourse on the resurrection, the body, at death, is said to be sown in corruption, in dishonour, in weakness, a mere natural and earthly body; flesh and blood cannot inherit the kingdom of God, neither doth corruption inherit incorruption: we shall be changed; this corruptible must put on incorruption, this mortal must put on immortality. In his second epistle he calls the body "an earthly house in which we groan, being burdened, earnestly desiring to be clothed upon with our house which is from heaven, that mortality might be swallowed up of life." In his epistle to the Romans, he gives the following comment on the Mosaic account of Adam's transgression; and its effects on the human race. "By one man sin entered into the world and death by sin, and so death passed upon all men for that all have sinned. Through the offence of one, many—that is, mankind at large—are dead. The judgment was by one to condemnation: by one man's offence death reigned and judgment came upon all men to condemnation. Our old man is crucified with Christ that the body of sin might be destroyed. He that is dead is freed from sin." He thus exhorts—"Let not sin reign in your mortal bodies that ye should obey it in the lusts thereof, nor yield your members as servants to unrighteousness." He speaks of "the infirmity of the flesh;" the motions, or, as in the margin, the passions of sin did work in our members to bring forth fruit unto death. In the seventh chapter he complains that he is "carnal, sold under sin. For that which I do I allow not, for what I would, that I do not, but what I hate, that do I. It is no more I that do it, but sin that dwelleth in me. In me —that is *in my flesh*—dwelleth no good thing, for to will is present with me, but how to perform that which is good I find not. The good that I would, I do not; but the evil which I would not, that I do. I find then a *law* that when I would do good, evil is present with me. *I delight in the law of God after the inward man,* but I see *another law in my members warring against the law of my mind,* and bringing me into captivity to the law of sin which is in my members. O, wretched man that I am! Who shall deliver me from the body of this death? or this body of death. So then with the *mind I myself serve the law of God;* but with the *flesh* the *law of sin,* the law of sin and death. The law was weak through the flesh. God sent his own Son in the likeness of sinful flesh. To be carnally minded is death. The minding, or disposition of the flesh, is enmity against God; it is not subject to the law of God, neither indeed *can* be—the body is dead because of sin—our mortal bodies. If we live after the flesh, we shall die, but if we through the Spirit do mortify the deeds of the body, we shall live. The creature was made subject to the bondage of corruption; we groan within ourselves, waiting for the adoption, to wit, the redemption of our body. Make no provision for the flesh to fulfil the lusts thereof. Those who cause divisions and offences serve their own belly." To the Ephesians he speaks of "the old man which is corrupt according to the deceitful lusts." To the Philippians he speaks of "our vile body, or the body of our humiliation." To the Colossians he represents the false teacher as "beguiling them in a voluntary humility and worshipping of angels, intruding into those things which he hath not seen, vainly puffed up by his fleshly mind—which things have indeed a show of wisdom in will worship and humility and neglecting of the body; not in any honour—to the satisfying of the flesh."

Nor are these opinions respecting the body, &c., peculiar to St. Paul. St. James declares that "every man is tempted, when he is drawn away of his own lust and enticed." He gives the following account of the origin and progress of sin. "Then when lust hath conceived, it bringeth forth sin: and sin when it is finished bringeth forth death. Envy and strife in the heart is earthly, sensual, and devilish. Wars and fightings come of the lusts which war in the members." St. Paul in his second epistle to Timothy speaks of certain "silly women laden with divers lusts and pleasures, ever learning, and never able to come to the knowledge of the truth:" of "those who will not endure sound doctrine, but after their own lusts heap up to themselves teachers, having itching ears, who turn away from the truth and shall be turned unto fables." St. Peter speaks of the "fleshly lusts which war against the soul;" he lays down the principle that he that hath suffered in the flesh, as Christ did, hath ceased from sin; speaks of "the corruption that is in the world through lust;" of "scoffers walking after their own lusts," and describes their scepticism. St. Jude predicts that mockers "should also so walk;" and speaks of "hating even the garment spotted by the flesh." It is also worthy of notice that the apostles describe the early heretics as peculiarly sensual and intemperate. Thus St. Peter portrays them as "counting it pleasure to riot in the daytime; having eyes full of adultery, or of an adulteress, walking in the lusts of uncleanness;" and St. Jude speaks of them as "feeding themselves without fear." In order to counteract the mental and moral alienation occasioned by voluptuousness, the primitive church enjoined fastings, vigils, &c. The Old Testament abounds with allusions to the physical causes of sin. It attributes Lot's incest to his intoxication, and Isaac's partiality for Esau, and consequent attempt to frustrate the divine appointment, to his love of his son's venison. Moses compares Jeshurun, the poetical name for Israel, to a pampered courser that "waxed fat, and kicked."* "Fatness of heart" is a usual metaphor for moral and religious insensibility; "great of flesh" is Ezekiel's description of a people abandoned to sensuality, and he adds, "this was the iniquity of Sodom: pride, fulness of bread and abundance of idleness, and they were haughty."† Solomon describes the infatuations and transgressions resulting from drunkenness,‡ and the extreme self-conceit of the sluggard.§ On the contrary, absence from wine, or strong drink, and eating any unclean thing, is prescribed, in one instance at least, as the instrumental means of a miraculous removal of sterility. Total abstinence from the produce of the vine, in any form, was enjoined on the Nazarites "who separated themselves unto the Lord."|| The Mosaic law prohibited articles of food unfavourable both to health and self-government, and was, in fact, a system of moral dietetics, in order that "Israel might be an holy people unto the Lord;" and circumcision itself was derived from moral reasons.¶ The foregoing quotations fully show that the Mosaic doctrine of the proximate cause of natural and moral evil among the descendants of Adam—originating in the change inflicted on Adam's physical nature and external circumstances, and which change was entailed on all his posterity, *for the purposes of their moral probation*—is uniformly

* Deut. xxxii. 15. † Chap. xvi. 26, 49, 50.
‡ Prov. xxiii.; comp. 1 Esdras iii. 17, &c. § Prov. xxvi. 16.
|| Judges xiii. 14; Num. vi. 2, &c.
¶ See Article, Circumcision, in the "Cyclopedia of Biblical Literature."

maintained from the commencement of the sacred canon to its close. That doctrine virtually includes the origin of even Eve's transgression; for, according to St. James, "lust," or desire, is the germ of transgression; but *all* unlawful desire, by whatever means *introduced* into the mind, *never*, as we know by consciousness, overcomes the moral powers but by means of a disturbance first raised by it in the physical part of our nature, and whereby the moral powers are for the time overwhelmed: and this origin of the first transgression, and of all its consequences, both shows the peril of forming even the incipient idea of any transgression, and is in perfect harmony with the statement of the Scriptures, that the salvation of the human race originated in the "pity of God" (Ep. to Titus ii. 3, margin); and further, by explaining the *modus operandi* of the original sentence, it also directs our attention to the sanitary and moral government of the body as one chief and essential means of human virtue and happiness: it also seems to reconcile us to the stern necessity of dying, and to endear to us the hope of a resurrection to a physical state of incorruption, moral "power," and "glory," in "a spiritual body"—teaches patience in regard of our own infirmities, and charity in regard of the infirmities of others, as well as submission to the limitations to our knowledge imposed on it by this "muddy vesture of decay."* In a word, the scriptural evidence now adduced shows us that the philosophy of physical circumstances is largely and decisively recognised by Revelation. But, what is more important to our present purpose, this doctrine affords incontestable support to the conclusion that every human mind, without exception, is liable, at least, to morbid influences, arising from its union with a disordered, mortal, and sinful body, even in its most healthy state, and under the most favourable external circumstances, and proportionably more so as the body is still further removed from its original state by disease, hereditary or incidental, chronic or temporary, occurring in the course of nature, or produced by some vice or mismanagement of the body, or by the reflex morbid effects upon it resulting from evil passions, ideas, &c. It shows also both the necessity and the practicability of constantly distinguishing between the pure perceptions of reason, or of that "mind with which we still serve the law of God," and those morbid influences of the body on the mind, and of avoiding all the means whereby the latter may be augmented. To use the words of Bishop Taylor, "Since it is our flesh and blood that is the principle of mischief . . . we must endeavour to abstain from those things which by a special malignity are directly opposed to the spirit of reason and the spirit of grace. . . . Nature is weak enough of itself, but these things take from it all the little strengths that are left to it, and then man can neither have the strengths of nature nor the strengths of grace."† Similar, too, is the doctrine of the ninth Article of the Church, which speaks of "the fault and corruption of the nature of every man, that naturally is engendered of the offspring of Adam, and is of his own nature inclined to evil, *so* that the flesh lusteth always contrary to the spirit, and this infection of nature doth remain even in them that are regenerated." The classical scholar will readily remember parallel references to the influence of the body on the mind, in Greek and Roman writers. The opinions, to the same effect, of the Catholic Fathers may be seen in the commentators on the Old and New Testament. Other subjects connected with the present can now only be alluded to,—such as the influence of the temperaments and external circumstances of the several authors of the Scriptures upon their writings, the diseases mentioned in the Scriptures, and their characteristic effects on the sufferers, as the elephantiasis of Job, Saul's melancholy, Naaman's leprosy, Nebuchadnezzar's zoanthropia, and the physical theory of demoniacal possessions.‡ It is hoped, however, that sufficient has now been advanced to

* *Merchant of Venice*, Act i., Sc. 5.

† Sermon on the Flesh and the Spirit.

‡ See Article, Demon, by the writer, in the "Cyclopedia of Bib. Lit."

demonstrate, by scriptural authority, the reality and importance of the subject of these papers, the remainder of which will be devoted to classifications of those co-existing morbid physical and religious phenomena which have come under the writer's notice. His wish to show that the doctrines of modern pathology are perfectly consistent with those of Revelation, and even highly illustrative of them, and to remove the suspicions with which the former are still too commonly regarded, must be his apology for the present digression from his principal design.

---

